# Xper-CT combined with laser-assisted navigation radiofrequency thermocoagulation in the treatment of trigeminal neuralgia

**DOI:** 10.3389/fneur.2022.930902

**Published:** 2022-08-02

**Authors:** Fengzhen Xiong, Tao Zhang, Qingbo Wang, Chenglong Li, Xin Geng, Qi Wei, Zhengbo Yuan, Zefu Li

**Affiliations:** Department of Neurosurgery, Binzhou Medical University Hospital, Binzhou, China

**Keywords:** trigeminal neuralgia, the new technology, laser navigation, Xper-CT, radiofrequency thermocoagulation

## Abstract

**Objective:**

Our objective was to study the clinical feasibility of Xper-CT combined with laser-assisted radiofrequency thermocoagulation in the treatment of trigeminal neuralgia.

**Materials and methods:**

A retrospective analysis was made of 60 patients with trigeminal neuralgia who visited the Affiliated Hospital of Binzhou Medical University from January 2019 to May 2021. According to the different surgical methods, we were divided into C-arm X-ray group and laser navigation group. The operation time, operative complications, post-operative 24 h, post-operative 3 and 6 months Barrow Neurotics Institute (BNI) score were recorded and compared.

**Results:**

Compared with the C-arm X-ray-guided puncture group, Xper-CT combined with laser-assisted navigation has the obvious advantages of shorter total puncture time, shorter surgical time, higher success rate of first puncture, and better surgical effect.

**Conclusion:**

Radiofrequency therapy of trigeminal neuralgia with Xper-CT combined with laser-assisted navigation has a good clinical effect and can be promoted and applied.

## Introduction

Trigeminal neuralgia (TN) refers to a severe pain that resembles an electric shock, a needle stick, a knife cut, or a tear in the distribution area of the facial trigeminal nerve (1). Long-term pain not only hurts the patient's body but also has a significant impact on the patient's mental and psychological state (2–4). It may even cause thoughts of suicide. At present, the most important theory about the pathogenesis of trigeminal neuralgia is the theory of local compression. Research believes that the pathogenesis of primary trigeminal neuralgia is due to the long-term compression of the trigeminal nerve by blood vessels, resulting in local demyelination of nerve fibers and mild irritation. It causes adjacent nerve fibers to “short-circuit” and accumulate repeatedly in pain neurons, causing intermittent, episodic severe pain. In addition, there are central lesion theory, virus infection theory and so on. According to the third edition of the International Headache Classification of Diseases (ICHD-3), trigeminal neuralgia can be divided into three categories: classical trigeminal neuralgia, secondary trigeminal neuralgia and idiopathic trigeminal neuralgia. Classical trigeminal neuralgia means that compressed nerves and vessels can be seen during MRI or surgery without obvious cause. In addition to clinical symptoms, organic diseases such as tumors, inflammation and vascular malformations can be found in clinical and imaging examination of secondary trigeminal neuralgia, and drug treatment is not effective, so it is usually necessary to treat the primary disease. Idiopathic trigeminal neuralgia refers to the typical symptoms of trigeminal neuralgia, but there is no obvious abnormality in nervous system examination.

With the continuous advancement of medical technology, the treatment methods for TN also tend to be diversified and include both drug and surgical treatment. For drug therapy, carbamazepine is now the first-line drug in the treatment of TN, but side effects can include somnolence, dizziness, liver damage, ataxia, and others (5, 6). Oxcarbazepine is also the first-line drug in the treatment of TN, which is well-tolerated and less likely to have drug interactions than carbamazepine. Other drugs, such as gabapentin, baclofen, and phenytoin, have also been shown to be effective (7, 8). Studies have also shown that for TN patients with severe oral pain, intraoral application of 8% lidocaine can produce immediate analgesia without serious side effects (9). However, for long-term relief, surgical treatment is more suitable for TN than drug treatment. There are many surgical treatments for TN, including radiofrequency thermocoagulation, semilunar ganglion balloon compression, stereotactic radiosurgery, and microvascular decompression. Radiofrequency thermocoagulation is a common surgical method for TN because of its decreased risk of trauma, no risk of craniotomy, relative safety for the elderly and people with other underlying diseases, and reliable initial and long-term effects (10, 11). However, when radiofrequency thermocoagulation is used in the treatment of TN, surgeons must accurately locate the oval foramen. Inaccurate needle location may not only lead to treatment failure but also to serious complications, such as bleeding, subarachnoid infection, masticatory muscle dysfunction, and intracranial neurovascular injury. Radiofrequency thermocoagulation was conceived to preferentially damage the small pain fibers. The electrode cannot be aimed at the first trigeminal division because the damage to small fibers entails corneal deafferentation and keratitis. Therefore, finding the accurate location is key to the success of radiofrequency thermocoagulation.

## Materials and methods

### Patient recruitment and selection criteria

This study included 60 patients with primary TN who were treated at the affiliated Hospital of Binzhou Medical College from January 2019 to May 2021. The clinical data of the patients are shown in Table 1. All patients were screened strictly according to the inclusion and exclusion criteria. This study has been approved by the Ethics Committee of Binzhou Medical College, and all patients gave written informed consent for this study.

**Table 1:** Characteristics of patients in C-arm X-ray group and laser navigation group.

**Characteristics**	**C-arm X-ray group (*n* = 30)**	**Laser navigation group (*n* = 30)**	***P*-value**
Age, year	67.23 ± 10.03	67.9 ± 8.38	0.787
Sex (male %)	11 (36.67%)	10 (33.33%)	0.787
Pain site (left side%)	9 (30.00)	14 (46.67)	0.184
Duration of TN (year)	4.52 ± 5.08	4.87 ± 4.35	0.605
Symptoms distribution (*n*)			
V1 only	2 (3.33)	2 (6.67)	0.326*
V2 only	7 (23.33)	6 (20.00)	
V3 only	3 (10.00)	6 (20.00)	
V1+V2	3 (10.00)	0 (0.00)	
V2+V3	15 (50.00)	15 (50.00)	
V1+V2+V3	2 (6.67)	1 (3.33)	
Pre-operative VAS score	8 (7.8)	8 (7.8)	0.93
First operation	24 (80.00)	26 (86.67)	0.488
Concomitant disease			
Hypertension	9 (30.00)	10 (33.33)	0.781
Diabetes	4 (13.33)	2 (6.67)	0.671*
Coronary heart disease (CHD)	3 (10.00)	1 (3.33)	0.612*
Cerebral infarction	3 (10.00)	2 (6.67)	>0.999*

^*^Fisher Accurate inspection.

The inclusion criteria were as follows: (a) those who are diagnosed as classical trigeminal neuralgia or idiopathic trigeminal neuralgia according to the third edition of the International Headache Classification of Diseases; (b) those who are ineffective or unable to tolerate the side effects of drugs after strict and standardized drug treatment; (c) those who have no mental disorders and are able to cooperate with the collection of clinical data; and (d) those who volunteered to join the trial. On the other hand, exclusion criteria were as follows: (a) those who do not cooperate with treatment, including those with mental disorders; (b) Those with an pre-operatively infection in the skin or deep tissue of the puncture site; (c) those with severe systemic diseases who cannot tolerate surgery; (d) those with blood clotting and bleeding disorders and tendencies before surgery; and (e) those with incomplete clinical data.

### Interventions

#### C-arm X-ray guided puncture

For each patient, the puncture point A was determined according to the branches of the diseased trigeminal nerve, and the puncture point was marked on the skin with a marker pen. Routine iodophor was used to disinfect the skin of the affected side and neck, sterile surgical drapes were spread on the skin, and 1% lidocaine local anesthesia was applied using the anterior approach, under the guidance of the C-arm X-ray, to determine the position of the needle. The radio frequency puncture needle enters slowly until near the oval foramen of the basicranial. When the puncture needle enters the oval foramen, the patient will experience severe pain. A side X-ray performed by a C arm is used to confirm the position of the needle tip. Connected with the radio frequency electrode, we chose 60, 65, and 70°C for 60 s for radiofrequency thermocoagulation (12, 13).

#### Xper-CT combined with laser-assisted navigation puncture

**Figure 1:**
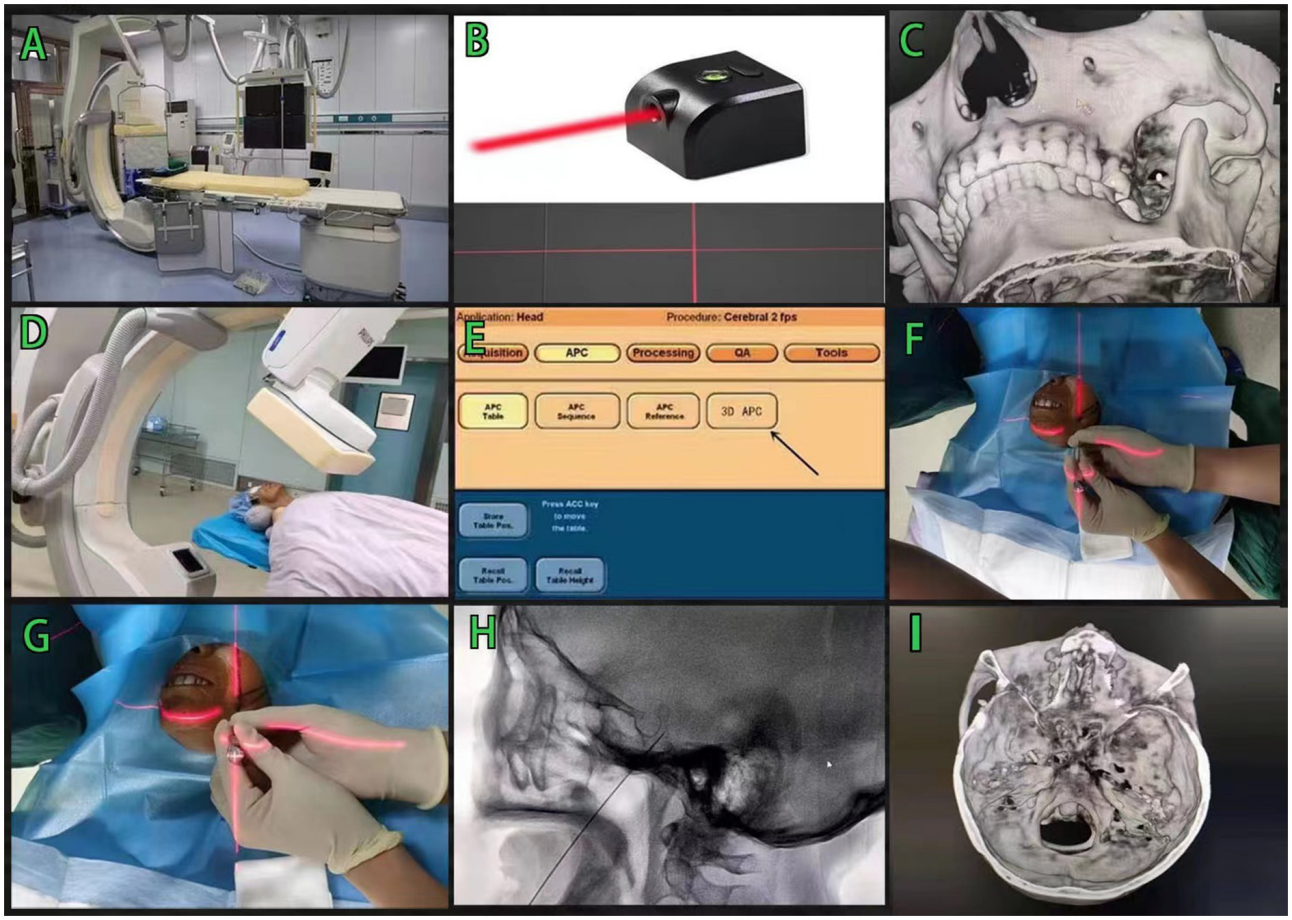
Laser navigation assisted DSA radiofrequency thermocoagulation in the treatment of trigeminal neuralgia. **(A,B)** Philip FD20 Digital Subtraction Angiography machine and cross-laser transmitter, **(C)** Adjust the 3D image so that the development line overlaps with the oval foramen, **(D,E)** Use the 3DAPC function in the Xper-APC automatic position control module to move the rack to a specified angle, **(F,G)** The puncture needle is punctured in the direction of the cross laser, and **(H,I)** The lateral X-ray of the head was taken to verify that the tip of the needle was in the oval foramen.

We used the Philip FD20 Digital Subtraction Angiography (DSA) machine and cross-laser transmitter for treatment (Figures 1A,B). According to the commonly used Hartel anterior approach, a medical developing colloidal barium thread with methylene silicone rubber as the main material is placed on the surface of the skin at about 2–3 cm outside the corner of the mouth, and its diameter is about 3 mm. We used a routine disinfection sheet with a 1% lidocaine local anesthesia. Using the Xper-CT three-dimensional (3D) reconstruction software of the DSA machine, the original data were collected for processing and 3D reconstruction, and the relationship between the puncture needle and the oval foramen position could be clearly observed in the reconstructed image. The reconstructed 3D image is rotated so the extended line overlaps with the oval foramen. Using the principle of “two points and one line,” the emission direction of the laser is determined, and the working angle of the 3D reference image is recorded (Figure 1C). We used the 3D Automatic Position Control (3D APC) function in the Xper-APC module to automatically adjust the rack to the angle shown by the 3D reference image (Figures 1D,E). We attached the bottom end of the laser transmitter to the Flat Detector (FD) so that the emitted laser is perpendicular to the FD. We moved the laser transmitter and aligned the cross laser focus to the puncture point. At this time, the direction of the laser emission will pass through the puncture point and the oval foramen at the same time, which is the direction of the puncture. During the puncture process, the focus of the laser is kept at the center of the needle tail of the puncture needle, and the puncture direction of the trocar will continue to face the oval foramen (Figures 1F,G). The lateral X-ray film of the head was performed by a DSA machine, and 3D reconstruction was used to verify whether the needle tip had been pierced into the oval foramen (Figures 1H,I).

### Clinical outcomes and follow-up

The therapeutic effects of the two groups were evaluated according to the intraoperative and post-operative conditions. Specifically, the operation time, the total puncture time and the success rate of the first puncture were used to evaluate the intraoperative effect. Indexes were used to evaluate the post-operative efficacy, such as facial numbness, keratitis and other post-operative complications. BNI scores were used to evaluate the long-term effects of the two groups at 24 hs, 3 and 6 months after operation.

### Statistical analysis

SPSS28.0 was used for data analysis. The measurement data for normal distribution were described by the mean plus and minus standard deviation, and the comparison between groups was described by two independent sample *t*-tests. The measurement data of the skewness distribution were described by median and quartile spacing, and the Mann–Whitney *U* test was used for intergroup comparison. The classified data were described by the number of cases and the rate. The chi-squared test or Fisher's exact test was used for the comparison between groups, and the Mann–Whitney *U* test was used for the comparison of grade data between groups. *P* < 0.05, it was considered that the difference was statistically significant. Repeated measurement data were analyzed by repeated measurement analysis of variance, and simple effect analysis was performed when the interaction was statistically significant. When the interaction was not statistically significant but the main effect should be statistically significant, the comparison between different time points in the group was compared by Bonferroni method. *P* < 0.05, it was considered that the difference was statistically significant.

## Results

### General pre-operative data

A summary of patients' demographic traits in the laser navigation and the C-arm X-ray groups, prior to therapy is outlined in Table 1. Summarily, we collected data from 60 patients with TN and divided them into laser navigation group and C-arm X-ray group according to the mode of operation, both of which were 30 patients. The average age of patients was not significantly different between the laser navigation (67.9 ± 8.38 years) and the C-arm X-ray (67.23 ± 10.03 years) groups (*P* = 0.781). Similarly, we found no significant differences in neither duration of TN between the laser navigation (4.18 ± 5.77 years) and the C-arm X-ray (4.87 ± 4.35 years) groups (*P* = 0.605), nor other parameters, including sex, pain site, symptoms distribution, pre-operative VAS score, and concomitant disease (Table 1).

### Comparison between intraoperative and post-operative efficacy

We compared efficacy of both methods, post-operation, and the follow up data is summarized in Table 2. Briefly, the operation time and total puncture time in the laser navigation group were lower than those in the C-arm X-ray group, whereas the number of successful cases of the first puncture in the laser navigation group was significantly higher than that in the C-arm X-ray group (*P* < 0.05). There was no significant difference in post-operative complications between the two groups (*P* > 0.05) (Table 2). The changes of BNI and time in C-arm X-ray group and laser navigation group were studied by single factor repeated measurement analysis of variance (ANOVA). The results showed that the main effects of group and time were significant (*p* < 0.05), and the interaction between group and time was also significant (F = 3.526 and *p* = 0.032 < 0.05). Then the interaction results were further analyzed by simple effect analysis. By comparing the BNI of the C-arm X-ray group and the laser navigation group at different times, we can see that the BNI of pre-operative in C-arm X-ray group is significantly higher than that of 24 h after, 3 months after operation and 6 months after operation, but there is no significant difference between 24 h after and 3 months after operation. The BNI value of 6 months after operation is significantly higher than that of 24 h after and 3 months after operation, and significantly smaller than that of pre-operative. In laser navigation group, the BNI of pre-operative was significantly higher than that of 24 h after, 3 months after operation and 6 months after operation, but there was no significant difference in BNI among 24 h after, 3 months after operation and 6 months after operation. In paired comparison with C-arm X-ray and laser navigation at four different times, the BNI value of C-arm X-ray in 24 h after group and 6 months after operation group was higher than that of Laser navigation, but there was no significant difference in other data (Table 3).

**Table 2:** The intraoperative and post-operative situation of C-arm X-ray group and laser navigation group.

**Intraoperative and post-operative condition**	**C-arm X-ray group (*n* = 30)**	**Laser navigation group (*n* = 30)**	***P*-value**
Operation time	60 (50.70)	45 (40.50)	<0.001
Total puncture time	10 (8.14)	4 (3.7)	<0.001
Successful puncture for the first time (*n*)	5 (16.67)	18 (60.00)	0.001
Post-operative complication			
Facial numbness	14 (46.67)	13 (43.33)	0.795
Keratitis	4 (13.33)	1 (3.33)	0.353*
Masticatory muscle weakness	2 (6.67)	0 (0.00)	0.492*

^*^Fisher Accurate inspection.

**Table 3:** The BNI score of C-arm X-ray group and Laser navigation group.

**Characteristics**	**Time**	**C-arm X-ray**	**Laser navigation**
		***M ±SD***	***M ±SD***
BNI	Pre-operative	4.267 ± 0.087	4.400 ± 0.087
	Twenty-four hours after	1.767 ± 0.118	1.333 ± 0.118
	Three months after operation	1.900 ± 0.162	1.600 ± 0.162
	Six months after operation	2.233 ± 0.185	1.700 ± 0.185

## Discussion

TN is a common cerebral nerve disease, and long-term pain causes harm to the physical and mental health of patients. The treatment of TN has been explored many years, resulting in more advanced treatments and a variety of options to choose from (14, 15). Microvascular decompression is the major surgical procedure for patients with classical TN with neurovascular conflict and morphological changes of the trigeminal root. One advantage it offers is that it can retain the anatomical integrity of the trigeminal nerve. As well, the normal nerve function of trigeminal nerve can be preserved after operation. With the continuous improvement of microsurgical technology, the incidence of surgical complications, such as hearing loss and facial sensory loss, has been greatly reduced (16, 17). However, it also has many limitations. First, the risk and high cost of the operation deter some patients. Second, it also requires an operator of excellent experience. Among the other surgical methods, radiofrequency thermocoagulation is effective in the treatment of TN, offering a simple operation with short operation time and low operation cost. It is especially suitable for patients with serious underlying diseases and cannot tolerate craniotomy or elderly and frail patients (18, 19). Compared with microvascular decompression surgery, the risk of repeated operation is lower, and it is more widely used (20).

The key of radiofrequency thermocoagulation is to accurately puncture the puncture needle into the oval foramen to accurately damage the semilunar ganglion of the trigeminal nerve and achieve the purpose of the treatment. However, under the traditional C-arm X-ray fluoroscopy, the puncture error is too large, and the surgeon has no reference for the puncture. The puncture is almost performed according to his own experience and feeling, which often requires multiple punctures, prolongs the operation time, and may also cause the patient irreversible damage (12, 21). At the same time, prolonged exposure to radiation can also cause damage to patients and doctors. With the development of technology, many auxiliary positioning methods have also appeared, such as CT-guided positioning, Neuronavigation-Assisted positioning of the oval foramen, and 3D printing guide-assisted positioning, which greatly improves the accuracy, improves the surgical effect, and reduces complications (22–25). However, the demand on the doctors' experience and equipment is high, and it is difficult to promote quickly. We used a simple laser instrument to coordinate with the DSA machine for positioning. After the DSA machine determines the puncture angle, we attach the laser instrument to the FD plate of the DSA machine. At this time, the laser instrument will emit a laser line, and this line is perpendicular to the FD plate. The laser instrument is moved to make the cross focus of the laser, coinciding with the puncture point on the patient's face. We determined the puncture direction and ensured that the focus is always kept at the center of the needle tail of the puncture needle during the puncture process so as to achieve the purpose of precise puncture.

Compared to C-arm X-ray guided puncture, Xper-CT combined with laser-assisted navigation puncture has varying advantages for the total puncture time, success rate of the first puncture, operation time, and other aspects, which can be considered to have a good clinical effect. The post-operative effect of the laser navigation group was also significantly better than that of the C-arm X-ray group. However, there are some shortcomings in this study. For post-operative complications, the sample size of this study is small, and there is no statistical difference in post-operative complications between the two groups. Second, whether it is a C-arm X-ray-guided puncture or a laser navigation-assisted DSA puncture, the incidence of facial numbness is very high, which is determined by the mode of the operation itself. The sensory branch of the trigeminal nerve is composed of different types of sensory nerve fibers. During the operation, it is difficult for operators to achieve the highly selective ablation of pain conduction fibers without damaging other sensory nerve fibers. This results in varying degrees of facial numbness after the operation. In addition, the patients included in this study are older, and most of them are female, there may be sample errors, and large-scale clinical trials are needed to further determine the specific results.

## Conclusion

Xper-CT combined with laser-assisted navigation radiofrequency puncture has a better clinical effect in the treatment of TN than ordinary C-arm X-ray-guided puncture. It offers greater advantages and a simpler operation and can be gradually popularized and applied in grassroots hospitals.

## Data availability statement

The original contributions presented in the study are included in the article/supplementary material, further inquiries can be directed to the corresponding author/s.

## Ethics statement

The studies involving human participants were reviewed and approved by Scientific Research Ethics Committee of Binzhou Medical University Hostipal. The patients/participants provided their written informed consent to participate in this study. Written informed consent was obtained from the individual(s) for the publication of any potentially identifiable images or data included in this article.

## Author contributions

FX designed and carried out the research, as well as wrote the article. ZL designed the research, performed report supervision, and revised the article critically for content. TZ, QWa, CL, XG, QWe, and ZY participated in the acquisition, analysis, and interpretation of the data and provided clinical advice. All authors contributed to the article and approved the submitted version.

## Conflict of interest

The authors declare that the research was conducted in the absence of any commercial or financial relationships that could be construed as a potential conflict of interest.

## Publisher's note

All claims expressed in this article are solely those of the authors and do not necessarily represent those of their affiliated organizations, or those of the publisher, the editors and the reviewers. Any product that may be evaluated in this article, or claim that may be made by its manufacturer, is not guaranteed or endorsed by the publisher.
